# EHMTI-0049. Headache, migraine and epileptic seizures

**DOI:** 10.1186/1129-2377-15-S1-D22

**Published:** 2014-09-18

**Authors:** N Gligic, S Atic, R Amanovic Curuvija

**Affiliations:** 1 Unit Stroke, Hospital for cerebrovascular diseases Sveti Sava, Belgrade, Serbia

## 

There are different possible temporal associations between epileptic seizures andheadache attacks wich have given rise to uncleare or controversal terminologies. Migraine endepilepsy have common pathophysichologic mechanisms and share essential anddefining attributes which distinguish them from other common neurologicaldisorders. They are both characterized by paroxysmal symptoms and episodic disorders. Occipital lobe to be thebrain structures most responsible for development of migraine and occipitallobe epilepsies Both are characterized by visual symptoms followed by headacheand other autonomic symptoms. Recognition of headache as an epilepticmanifestation per se still represents a challenge.

The classification of the international league AgainstEpilepsy does not refer to this type of disorder, while the International Classification of headache Disorderdefines kinds of association _ migraine triggered seizures hemicrania epilepticaand post ictal headache.

Epileptic headache or ictal epileptic headache is anepileptic manifestation per se, with onset and cessation if isolated ,coinciding with EE patern of an epileptic seizures EH maybe followed by other epleptic i manifestation /motor/ sensory/ autonomic/.

Hemicrania epileptica is very rare variant . Post ictalor pre ictal headache _ when headache is followed during or short time aftertypical epi seizures Migraine attac maybe with or without aura, and seizures triggerring role is stole a subjectdebate.

No conflict of interest.

